# Stem Cells: Innovations in Clinical Applications

**DOI:** 10.1155/2014/516278

**Published:** 2014-07-07

**Authors:** Morgan T. Sutton, Tracey L. Bonfield

**Affiliations:** ^1^Department of Pediatrics, Case Western Reserve University, Cleveland, OH 44106-4948, USA; ^2^Hathaway Brown School, 19600 North Park Boulevard, Shaker Heights, OH 44122, USA

## Abstract

The use of mesenchymal stem cells (MSCs) as clinical therapeutics is a relatively new avenue of study for treatment of a variety of diseases. The therapeutic impact of the MSCs is based upon their multiplicities of function and interaction with host tissues. MSCs can be anti-inflammatory, antifibrotic, antimicrobial, and regenerative, all which may improve outcomes in scenarios of damaged tissues and inflammation. Although most studies focus on utilizing MSCs to direct clinical efficacy, it is the ability to orchestrate host response in surrounding tissue that is especially unique and versatile. This orchestration of host response can be applied to a variety of clinical scenarios not only through cell-cell interactions but also through production of bioactive secreted factors. These bioactive factors include small proteins, chemokines, cytokines, and other cellular regulators. These factors have the capacity to induce angiogenesis or blood vessel development, be chemotactic, and induce cellular recruitment. MSCs also have the capacity to differentiate with the implicated environment to regenerate tissue or accommodate host tissue in a cell specific manner. The differentiation cannot only be done *in vivo* but also can be optimized *in vitro* prior to *in vivo* administration, potentiating the versatility of the MSCs and opening avenues for corrective therapy and cell delivery of genes. The differentiation process depends on the environment with which the MSCs are put and results in active communication between the newly administered cells host tissue. Since these properties have been identified, there are a variety of clinical trials and studies being conducted on MSCs ability to treat human disease. This review outlines the potential use of MSCs, the types of tissue, and the innovative applications of MSCs for the treatment of diseases.

## 1. Introduction

Mesenchymal stem cells (MSCs) are multipotent cells that secrete a variety of bioactive factors, which actively contribute to their environment. These cells are also capable of changing to suit their environment, being responsive to host tissue cues. These tissues can be diverse ranging from bone, cartilage, lungs, pancreas, the central nervous system, the gastrointestinal track, and the circulatory system [1]. These properties could be helpful in scenarios of tissue damage, inflammation, and infection associated with these organs implicating the power of MSCs therapeutic potential: versatility and applicability. However, from a research standpoint, this property can be conflicting, as the impact of MSCs themselves is still controversial. The issue begins with the unknowns, as it has not been concluded whether the improvement in the damaged tissue or area of inflammation and infection is because of the MSCs or whether the improvement is caused by the tissues' response to the MSCs or both [2]. This review will give insight into the research and clinical trials that are being done to determine the efficiency of stem cells in a host of different environments, as well as new avenues for patient care.

## 2. Stem Cells in the Treatment of Wounds

**Figure 1:**
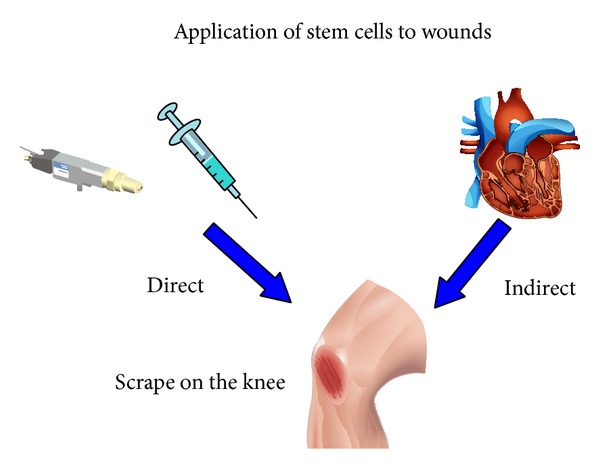
The application of stem cells in wound treatment can be done directly with injection or topical application or indirectly through systemic administration. Direct application of MSCs to the effected region is more effective in the treatment of wounds with a significantly faster response time to the MSCs and minimization of lost therapeutic impact.

Wound healing is a complex process that involves mitosis, inflammation, angiogenesis, synthesis, and remodeling of the extracellular matrix [3]. When wound healing does not occur, the wound may become chronic and need additional interventions [3]. MSCs are very versatile and promote pro- and anti-inflammatory responses, along with angiogenesis [1]. Research has been performed on the effects of using MSCs in the treatment of wounds, both with indirect and direct delivery to the wound site (Figure 1). With indirect delivery, the MSCs are infused systemically into the circulatory system [4]. New studies have shown that MSCs home to sites of injury and provide therapeutic impact [4]. Several studies have suggested that MSCs home to regions of injury by specific trafficking to chemokine ligand 7 (CCL7) [5, 6]. Once the MSCs reach the point of injury, the MSCs exit the vasculature in the connective tissue stromal region [4]. The MSCs respond to the specific tissue milieu while at the same time contribute to the milieu through the secretion of biomolecules [7]. This exquisite interaction between the tissue and the MSCs defines the efficacy, potency, and overall therapeutic impact of the MSCs. Further, the MSCs can become continuous resources for sustaining the tissue milieu of the therapeutic impact. The issue with the intravenous delivery of the MSCs is localization in the lungs. This latter fact has been the reasoning behind using MSCs for treating lung diseases associated with unrelenting or acute inflammation such as asthma and cystic fibrosis (see later in this text). Even with the initial distribution of the MSCs in the lungs, in the majority of studies MSCs are difficult to identify after a week or so. This is due to the hypothesized localization of the MSCs to tissue sites of damage with subsequent migration to the tissue of destination. The issue with using indirect delivery is the risk that the MSCs may go off route in the spleen, liver, and lungs, and if the designated site for impact is not in these areas there may be a significant decrease in therapeutic impact. This derouting of the MSCs slows their movement to the site of injury and decreases the number of MSCs that are present at the site of injury. Although this allows for the flexibility of MSC action, it does potentially result in dilution of the MSC impact. Recently, new directions in optimizing the therapeutic application of MSCs at their site of impact have become an exciting avenue for researchers. This involves direct application of MSCs to the wounded area [4]. These methods would include direct injection into the wound site as seen in the new models of urinary incontinence, arthritic lesions, and a variety of neuronal diseases [7]. To use this method, the MSCs must be injected adjacent to the wound site, or they must be placed directly onto the site of injury [8]. In a research study, Stoff and his colleagues found that human MSCs injected near the site of injury in immunocompetent rabbits improved tissue function and reduced the amount of scarring [9]. Further, Stoff found that there was no evidence of rejection of the MSCs.

In another study by Dr. Falanga et al. , MSCs were placed directly on the site of injury resulting in wound improvement [10]. In similar experiments performed by Dr. Stoff et al., it was shown that the use of fibrin MSCs sprayed in wounds caused the injury site to heal much faster and showed a more progressed histology than those wounds not treated with topical MSCs [4, 10]. The results from the application of MSCs to wound areas have opened the door to study applications of MSCs immunomodulatory potential toward wound healing and injury. Previous studies have shown that MSCs can be activated using cytokines such as granulocyte colony stimulating factor (GM-CSF), tumor necrosis factor (TNF-*α*), or interferon gamma (IFN*γ*) to enhance activity and therapeutic impact. In these studies it has been shown that when MSCs are activated with IFN*γ*, the MSCs secrete soluble factors, which enhance killer T cells and early stage dendritic cells [4, 11]. It could be argued that manipulation of the MSCs by the cytokines may provide ways in which MSC function can be augmented for specific applications. Depending on the disease or specific scenario, MSCs could be made to be even more potent in terms of application. Further, the soluble factors generated by MSCs may also provide new and innovative directions in cell therapy beyond treating wounds and in commercialization of identified products [4, 12].

## 3. Stem Cells in Orthopedics

**Figure 2:**
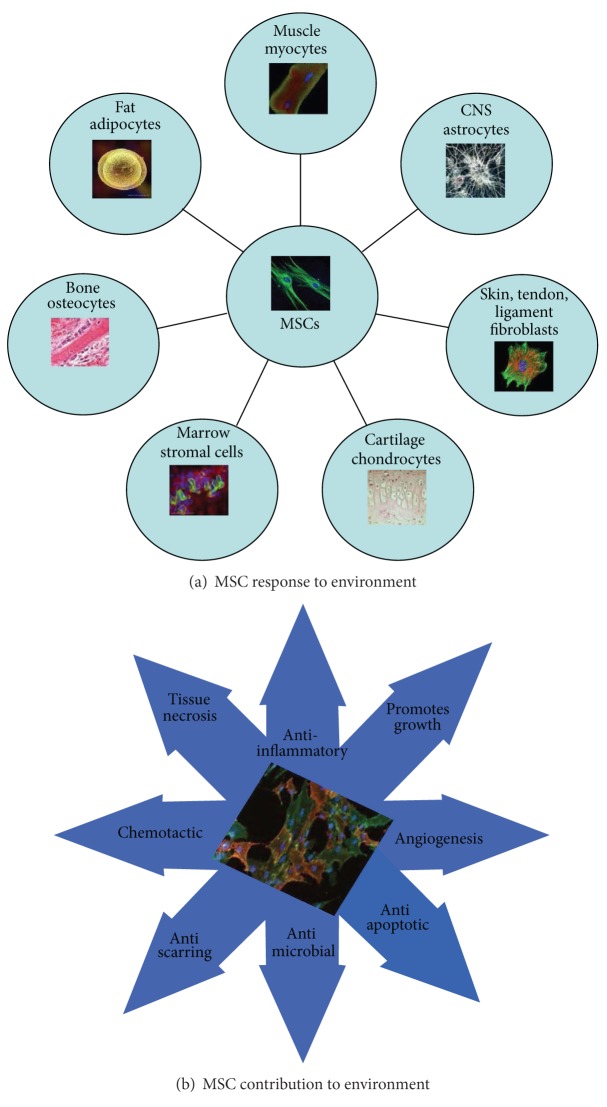
MSCs in environment. MSCs are both responsive and contributive to their environment. MSCs have the ability to differentiate into multiple cell types (a), which also contribute to their environment. In the orthopedic world, MSCs have the ability to transform into a regeneration of bone, cartilage, bone marrow, muscles, tendons, and connective tissue, as shown above (a). MSCs secrete healing products, which contribute greatly to their environment (b).

It is known that MSCs have the capability to move chemotactically to areas of inflammation and infection in an organism's tissue [13]. MSCs also secrete a multitude of cytokines which exhibit anti-inflammatory mechanisms in the microenvironment as seen in Figure 2 [13]. MSCs actively contribute to tissue regeneration such as intravenous routes, secreting soluble factors, and regulating inflammatory responses [13]. Further, MSCs also secrete factors which promote bone regeneration (Figure 2). The use of MSCs in an orthopedic setting has promising outcomes due to these factors. In cartilage regeneration, MSCs have been used as a therapeutic to repair damage. In research conducted by Shafiee and colleagues, MSC based cartilage repair in rabbit models that had full thickness cartilage defects showed promise with improved healing, measured through macroscopic scores [14]. At six months after the study, the MSCs showed effectiveness in chondrocyte transplantation, as well as tissue regeneration [14]. The results showed a significant improvement in overall clinical score, although there was no complete hylane cartilage detected [15]. In studies by Scott et al, cellular allografts containing MSCs were used in high-risk foot and ankle reconstructions [15]. MSCs have been used to enhance ostoconstruction* in vivo*, because of their osteogenic potential. In these studies stem cell were grafted in hindfoot and ankle surgery which improved healing and interval to partial weightbearing. These studies implicate the use of cellular allografts containing MScs in foot and ankle surgery [15].

The largest problems are big bone defects, often as a result of infection, tumors, trauma, insufficient blood supply or as a post-infection consequence [16]. These defects pose a great problem, as bone supply is greatly limited, thus creating difficulties in producing autologous bone grafts [16]. These bone grafts also result in high levels of morbidity in donors, as well as a heightened risk of disease transmission or rejection in recipients [16]. Thus the use of MSCs as an alternative treatment in the area of bone defects is appealing. In a mouse model performed by Granero-Molto, bone marrow MSCs showed movement toward the site of fracture to begin regeneration after systemic application of MSCs [17]. The model also demonstrated that the bone marrow MSCs enhanced tissue healing in the fracture site and actively contributed to a significant decrease in the amount of inflammation both locally and systemically [16, 17]. Further, the MSCs differentiated into bone cells at the site of fracture, promoting the production of angiogenesis by paracrine factors [16, 17]. In an elegant study by Lin et al. [18] using luminescence and fluorescently tagged MSCs, they showed that regardless of how the MSCs are administered, they localize to areas of injury including bone damage. The interesting aspect of these studies is that with time the MSCs became less dense but had the capacity to localize to the bone injury followed by some regenerative capacity [19]. Although these studies implicate the use of bone marrow MSCs in bone regeneration and orthopedic applications there is still work that is needed in terms of improving wound healing by developing new and innovative scaffolds and enhancing the production of soluble mediators.

## 4. Stems Cells in Hematological Pathology

Hematopoietic stem cells (HSCs) are often used as a treatment in hematological pathologies but can cause many adverse reactions, such as bleeding, graft versus host disease (GvHD), and other forms of rejection [19]. MSCs have the potential to successfully aid in HSC engraftment and prevent rejection with their immune-suppressive properties. MSCs also generate cytokines that aid hematopoiesis and could enhance the efficacy of MSCs in bone marrow recovery after chemotherapy and/or radiation [19]. In a study performed on a patient who had severe idiopathic aplastic anemia, MSCs were infused in combination with HSCs for use as a possible therapeutic. After MSC/HSC infusion, most of the ensuing medical problems were resolved, although there was still no recovery of hematopoietic tissue [19]. This study indicated that MSCs had the potential to be a safe addition for use as a coinfusion cellular therapeutic with HSCs [19]. The studies were further evidence for MSC use in hematopoietic pathology as a phase I clinical trial. The trial resulted in hematopoietic recovery for most patients, with 50% of patients not developing GvHD [19, 20]. These studies suggest that introducing culture expanded MSCs with the HSCs for transplantation could be an effective and safe process that could minimize the side effects and facilitate bone marrow recovery [20].

## 5. Stems Cells for Inherited and Neurological Diseases

MSCs have shown their versatility in multiple situations [19]. Further, MSCs have demonstrated their ability to change into neurons and astrocytes [21]. Due to these observations, mouse models have been used to test MSC transplants on mice with acid sphingomyelinase, a neurodegenerative disease. Infusion of the MSCs resulted in a delay in the start of neurological abnormalities and improved overall survival in the mouse model [19]. Based on this experiment, a study was started to determine the effectiveness of MSC transplantation into human patients with amylotrophic lateral sclerosis, a disease that causes degeneration of motor neurons and muscle functionality [19]. Using bone marrow aspirates from each of 7 patients, MSC cultures were prepared over the course of 3-4 weeks. After injection of the MSCs into the spinal cord of the patients, magnetic resonance imaging (MRI) was performed at 3 and 6 months [21]. A slow trend of improvement in muscle strength was detected, although there was not enough preliminary data to conclude on how long the direct effect could be sustained.

Central nervous system injury (CNS) situations can be caused by a stroke, trauma, or an underlying neurological condition. In CNS, neural MSCs (NSCs) and MSCs are used for regeneration purposes to create new cells to replace those that were lost [22, 23]. However, this process has not been completely effective due to oxidative stress and toxic by-products, which can affect MSC transplantation [23, 24]. This causes slowing of tissue regeneration, as well as reduced longevity. Currently, carbon nanotubules (CNTs) are being used to support MSC differentiation in the area of nanomedicine. In these studies CNT/MSC composites were used to improve neurite growth after CNS damages. In both the* in vivo *and* in vitro *settings, the study demonstrated biocompatibility of the CNTs with MSCs and NSCs [23]. This observation could direct neuron function and promote healing of damaged neural tissue [22, 23]. In yet another neurological disease, Parkinson's, MSCs have been shown effective at inhibiting inflammatory cytokine production, a main factor that contributes to the disease. Scientists from the University Hospital of Tubingen in Germany observed yet another effective way to deliver MSCs to neurological patients, through the nose. Studies were performed in a Parkinson's induced rat model, with intranasal administration of bone marrow MSCs [24]. In these rodents, MSCs were found in the olfactory bulb, cortex, striatum, cerebellum, brain stem, hippocampus, and spinal cord up to 4.5 months after administration, providing data that suggested MSCs could proliferate* in vivo *successfully. It was observed that intranasal administration increased tyrosine hydroxylase levels in the lesioned ipsilateral striatum and substantia nigra, while decreasing levels of the toxin 6- hydroxydopamine [24]. Decreases in TNF*α*, interleukins (IL) 2, 6, and 12, and IFN*γ* were observed in an association with cell therapy [24]. This method of intranasal administration could change the face of MSC administration [24].

In situations of genetically inherited diseases, bone marrow MSCs have also been used as a therapeutic for Hurler's syndrome and metachromatic leukodystrophy. After undergoing successful bone marrow transplantation from human leukocyte antigen (HLA) identical siblings, 11 patients suffering from metachromatic leukodystrophy were given bone marrow MSCs from their sibling donors by injection. In four of the patients, there was significant improvement in nerve conduction velocities [24, 25]. More studies need to be done, however, before success or failure of bone marrow MSCs can be determined in situations of inherited diseases [25].

## 6. Stem Cells in Diabetes

**Figure 3:**
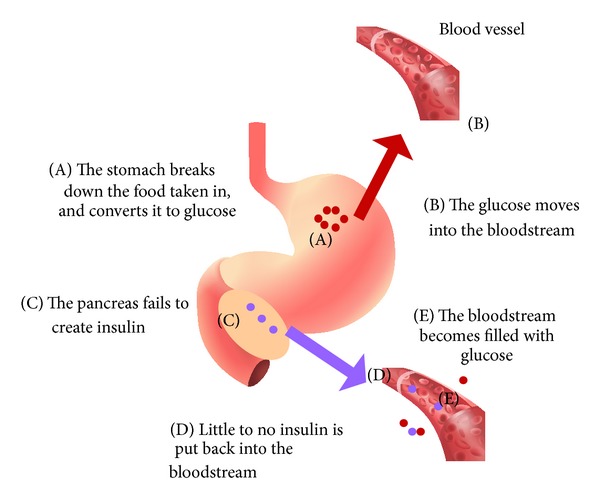
Diabetes and insulin. In the digestion of food, a diabetic patient does not create insulin, causing a glucose buildup in the bloodstream and an elevated blood sugar level.

Diabetes is defined by a person's inability to maintain proper blood insulin levels (Figure 3). With the shortage of insulin producing cells in diabetes, pancreas and inslet transplantations have been performed to eliminate the need for insulin injections on a regular basis [27]. The issue is that pancreas and inslet cells are scarce and are often rejected by the recipient after implantation. Thus, the use of embryonic stem cells (ESCs) has been pursued as a way to generate insulin producing cells, or beta cell surrogates to overcome these issues. The use of ESCs can be an ethical issue, along with a high rate of rejection after use [27]. From these implications, autologous stem cells (ASCs) become a good alternative because they eliminate the risk of rejection without the ethical stigma of ESCs. One of the most attainable sets of ASCs would be peripheral blood, which also contains the normal human insulin producing cells [25, 27]. These cells can be isolated easily from autologous blood based on their phenotype. Through experiments performed by the Zhao Laboratory [27], it was found that peripheral blood insulin producing cells could be isolated and preserved for future insulin production because they have the ability to hinge onto a polystyrene petri dish, and they showed transcription and insulin production at protein and mRNA levels [27]. This technology would allow patients to generate their own insulin producing cells [27]. This treatment would eliminate the hazard of rejection by the immune system, shorten the time to transplant due to the shortage of donors, and would have no ethical issues. A clinical trial performed by Dr. Voltarelli [28] on newly diagnosed type 1 diabetes patients showed prolonged insulin independence in most participants after transplantations with HSCs [28]. This is not the only application of MSCs in diabetes. As has been discussed in the previous sections, MSCs can also be used in scenarios associated with the defective wound healing and diabetic neuropathy [29]. These observations suggest a significant impact of MSCs in the treatment of this disease which may provide better avenues for patient care.

## 7. Stem Cells in Lung Diseases

**Figure 4:**
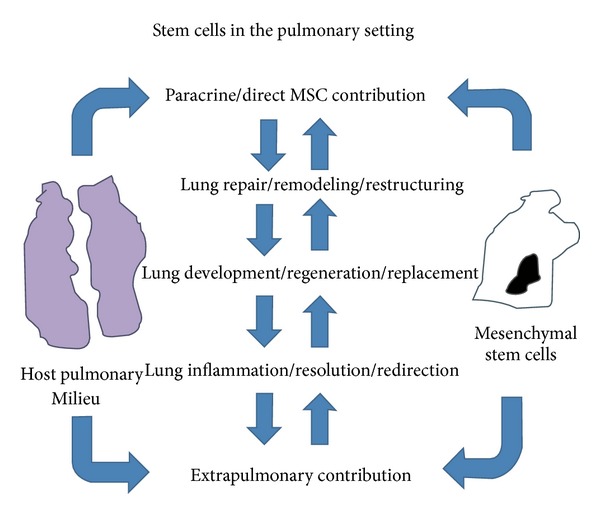
In the setting of chronic lung disease, MSCs have great potential to be an alternative therapeutic. MSCs can contribute to lung regeneration and reduction of inflammation, as well as improve fluid clearance Bonfield et al. [26].

MSCs have the potential to impact damaged or inflamed lung areas by repairing the tissue or stimulating the host tissue to regenerate itself. In lung conditions involving fibrotic disease, MSCs would be involved in reversing extracellular matrix deposition and collagen synthesis modeled in Figure 4 [26, 30, 31]. In the situation of idiopathic pulmonary fibrosis (IPF), lung fibrosis results in scarring and terminal pulmonary insufficiency as seen in Figure 4 [29, 31, 32]. In a study performed on a bleomycin model, which shows similar morphology to IPF, bone marrow MSC administration following bleomycin treatment displayed a decrease in both collagen deposition and in inflammation [31–33]. In another study, it was found that murine MSCs home to the lung in response to injury and become epithelium like in phenotype while decreasing lung tissue inflammation [31, 33–35]. Acute lung injury (ALI) is a devastating disease with a high mortality rate and significant morbidity [7, 36]. Injury to alveolar epithelium, vascular endothelium, and endotoxins are common effects. Treatment with MSCs decreased pro-inflammatory cytokines, whereas the resolution response and anti-inflammatory cytokines levels increased [33, 37]. Further, mice given murine MSCs had decreased levels of alveolar capillary permeability, extravascular edema, and mortality [33]. In a placebo controlled study of MSCs in patients suffering from Chronic Obstructive Pulmonary Disease (COPD), characterized by severe lung and systemic inflammation, MSCs were infused intravenously [34]. Patients showed early, significant decreases in circulatory reactive protein (CRP) with MSCs treatment, creating a solid foundation for continuing clinical trials of MSCs for COPD [34] endotoxin induced lung injury [24, 33, 37]. These studies suggest that the use of MSC in the treatment of ALI, COPD, and IPF could be a therapeutic option.

## 8. Stem Cells and Cystic Fibrosis

Cystic fibrosis (CF) is a genetically inherited disease which results in mutations in the cystic fibrosis transmembrane regulator (CFTR) gene. The mutation in this disease impacts almost every organ of the body, but the major cause of morbidity and mortality is the inability to control lung infection and inflammation. Since bone marrow MSCs have both anti-inflammatory and antimicrobial properties studies were done to investigate the potential of using MSCs as a therapeutic in the murine model of CF lung infection and inflammation [1, 2, 38]. In this model CF mice lose considerable weight without resolution and often succumb to the infection. Therapeutic bone marrow MSCs in this model resulted in weight changes similar to control mice with improved gross lung pathology and decreased cellular recruitment into the lung. Further, the bone marrow MSCs shifted the pulmonary differential from predominantly neutrophils to a more evenly distributed differential of both neutrophils and macrophages [38]. Importantly, even though the inflammatory profile was decreased in severity, there was no increase in bacterial load. These studies have provided the first series of preclinical data to support the potential of using MSCs as a new cell based therapeutic intervention in CF [2, 26].

## 9. Stem Cells in Allergy and Asthma

**Figure 5:**
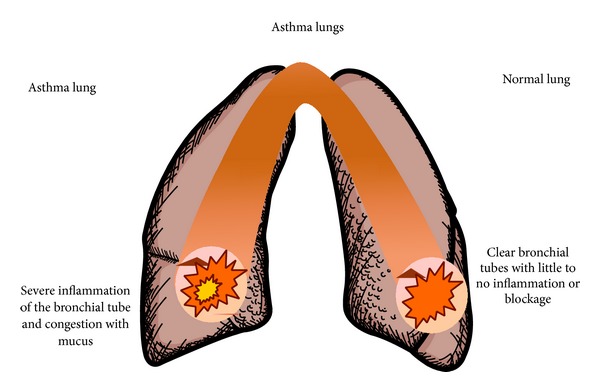
Asthma causes severe inflammation of the bronchial airways, making it hard to breathe. The use of MSCs as a therapeutic in this scenario caused a significant decrease in fluid blockage, as well as reduced the inflammation in the airways.

Asthma is a chronic inflammatory disease that causes airway inflammation and reactivity, which can ultimately result in lung injury modeled in Figure 5 [26, 38, 39]. MSCs have anti-inflammatory and growth promoting mechanisms that make them an appealing therapeutic for chronic asthma. Observations published by the Weiss laboratory showed that when the ovalbumin asthma model was given murine MSCs there was a significant decrease in airway hyper-responsiveness and eosinophil levels in bronchoalveolar lavage fluid (BAL) [35]. This also influenced the direction of T-cell response, shifting away from helper cell (Th2) cytokines [35]. Other studies have also shown that the MSCs given to the mice decreased levels of epithelial hyperplasia, inflammation, and extracellular matrix deposition. There were no adverse side effects detected in these models, even though human MSCs were used in mice [33, 39].

The immunomodulatory properties of MSCs provide new avenues of therapeutic potential to treat allergy. In a study performed by the Sun laboratory, mice were given allergic inflammation in their upper and lower airways. With the use of murine MSCs, the mice showed inhibited nasal eosinophilia and lung pathology [40]. With the suppressed pathology and balancing of immune response, inflammation was significantly reduced in both the upper and lower airways in the model, after challenge with MSCs [40]. Bone marrow transplantation in allergy has resulted in both transfer of the allergy to the recipient as well as prevention of allergy in the recipient [41]. This may be relevant to the hematopoietic cell source. It has been shown that bone marrow stromal cells inhibit mast cell function via cyclooxygenase 2 (COX-2) dependent mechanisms which suggest that there may be potential applications of bone marrow MSCs toward the treatment of mast cell inflammatory disease, such as anaphylaxis [41], but the source of MSCs may be important in these studies. These observations lend themselves to the potential of using MSCs as an alternative cell source for the treatment of severe allergic disease.

## 10. Summary

Mesenchymal stem cells (MSCs) have a promising future in the world of clinical medicine. With their ability to differentiate into many cells types and their sensitivity to their environment MSCs have the potential to be a useful therapeutic for many areas of medicine. Clinical trials are ongoing to assess MSC ability in many diseases, (http://www.clinicaltrials.gov/). The MSCs versatility in any environment has made them an attractive resource for disease treatment, though the technology and full understanding of MSC mechanisms are still in their preliminary stages. The application of MSCs in clinical medicine is sure to become the front-runner in innovative therapeutics for the research and the clinical setting.
